# 9-Oxo-4,5-diaza­fluoren-4-ium tetra­chloridoaurate(III)–4,5-diaza­fluoren-9-one (1/1)

**DOI:** 10.1107/S1600536809006734

**Published:** 2009-02-28

**Authors:** Nasser Safari, Vahid Amani, Behrouz Notash, Seik Weng Ng

**Affiliations:** a Department of Chemistry, General Campus, Shahid Beheshti University, Tehran, Iran; b Department of Chemistry, University of Malaya, 50603 Kuala Lumpur, Malaysia

## Abstract

The Au^III^ atom in the title compound, (C_11_H_7_N_2_O)[AuCl_4_]·C_11_H_6_N_2_O, is in a nearly square-planar environment defined by four Cl atoms. The protonated 9-oxo-4,5-diaza­fluoren-4-ium cation forms an N—H⋯N hydrogen bond with the neutral 4,5-diaza­fluoren-9-one mol­ecule.

## Related literature

For other 9-oxo-4,5-diaza­fluoren-4-ium tetra­chlorido­metal­lates, see: Kulkarni *et al.* (2003 ▶); Menon *et al.* (1994 ▶); Ravikumar *et al.* (1995 ▶); Ravikumar & Lakshmi (1994 ▶); Zhang *et al.* (2003 ▶). For the synthesis of 4,5-diaza­fluoren-9-one, see: Henderson *et al.* (1984 ▶).
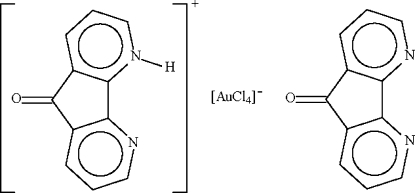


## Experimental

### 

#### Crystal data

(C_11_H_7_N_2_O)[AuCl_4_]·C_11_H_6_N_2_O
*M* *_r_* = 704.13
Triclinic, 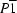

*a* = 7.1035 (1) Å
*b* = 12.6513 (2) Å
*c* = 13.2366 (2) Å
α = 73.285 (1)°
β = 78.410 (1)°
γ = 88.375 (1)°
*V* = 1115.51 (3) Å^3^
*Z* = 2
Mo *K*α radiation
μ = 7.10 mm^−1^
*T* = 118 K
0.20 × 0.10 × 0.10 mm

#### Data collection

Bruker APEXII CCD diffractometer
Absorption correction: multi-scan (*SADABS*; Sheldrick, 1996 ▶) *T*_min_ = 0.331, *T*_max_ = 0.537 (expected range = 0.303–0.491)
9343 measured reflections
5044 independent reflections
4829 reflections with *I* > 2σ(*I*)
*R*_int_ = 0.016

#### Refinement

*R*[*F* ^2^ > 2σ(*F* ^2^)] = 0.025
*wR*(*F* ^2^) = 0.067
*S* = 1.04
5044 reflections
302 parameters
1 restraint
H atoms treated by a mixture of independent and constrained refinement
Δρ_max_ = 3.05 e Å^−3^
Δρ_min_ = −1.27 e Å^−3^

### 

Data collection: *APEX2* (Bruker, 2007 ▶); cell refinement: *SAINT* (Bruker, 2007 ▶); data reduction: *SAINT*; program(s) used to solve structure: *SHELXS97* (Sheldrick, 2008 ▶); program(s) used to refine structure: *SHELXL97* (Sheldrick, 2008 ▶); molecular graphics: *X-SEED* (Barbour, 2001 ▶); software used to prepare material for publication: *publCIF* (Westrip, 2009 ▶).

## Supplementary Material

Crystal structure: contains datablocks global, I. DOI: 10.1107/S1600536809006734/hy2184sup1.cif

Structure factors: contains datablocks I. DOI: 10.1107/S1600536809006734/hy2184Isup2.hkl

Additional supplementary materials:  crystallographic information; 3D view; checkCIF report

## Figures and Tables

**Table 1:** Selected bond lengths (Å)

Au1—Cl1	2.2720 (8)
Au1—Cl2	2.2882 (8)
Au1—Cl3	2.2864 (8)
Au1—Cl4	2.2872 (8)

**Table 2:** Hydrogen-bond geometry (Å, °)

*D*—H⋯*A*	*D*—H	H⋯*A*	*D*⋯*A*	*D*—H⋯*A*
N1—H1⋯N3	0.89 (1)	1.88 (1)	2.762 (4)	168 (4)

**Fig. 1.**
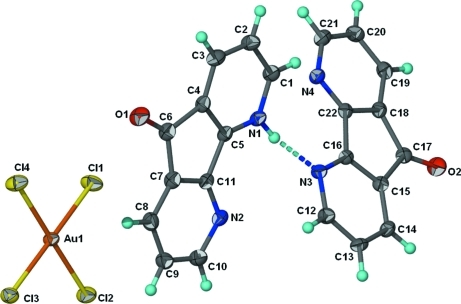
Molecular structure of the title compound. Displacement ellipsoids are drawn at the 70% probability level. The dashed line denotes hydrogen bond.

## supplementary crystallographic information

### Experimental

Chloroauric acid trihydrate (0.25 g, 0.73 mmol) dissolved in acetonitrile (5 ml) was mixed with 4,5-diazafluoren-9-one (Henderson *et al.*, 1984) (0.13 g, 0.73 mmol) dissolved in methanol (5 ml). The yellow solution was set aside for the growth of crystals, which appeared after two weeks (yield 70%; m.p. 483 K).

### Refinement

C-bound H atoms were positioned geometrically and refined as riding atoms, with C—H = 0.95 Å and with *U*_iso_(H) = 1.2*U*_eq_(C). The N-bound H atom was located in a difference Fourier map and refined isotropically, with a distance restraint of N—H = 0.88 (1) Å. The crystal diffracted strongly owing to the extremely heavy metal atom; however, its presence introduced severe absorption problems that could not be corrected analytically as the crystal did not have regular faces. The final difference Fourier map had a highest peak at 1.00 Å and a deepest hole at 1.00 Å from Au1 atom.

### Crystal data

**Table:**

(C_11_H_7_N_2_O)[AuCl_4_]·C_11_H_6_N_2_O	*Z* = 2
*M**_r_* = 704.13	*F*(000) = 672
Triclinic, *P*1	*D*_x_ = 2.096 Mg m^−^^3^
Hall symbol: -P 1	Mo *K*α radiation, λ = 0.71073 Å
*a* = 7.1035 (1) Å	Cell parameters from 7741 reflections
*b* = 12.6513 (2) Å	θ = 2.7–28.4°
*c* = 13.2366 (2) Å	µ = 7.10 mm^−^^1^
α = 73.285 (1)°	*T* = 118 K
β = 78.410 (1)°	Block, yellow
γ = 88.375 (1)°	0.20 × 0.10 × 0.10 mm
*V* = 1115.51 (3) Å^3^	

### Data collection

**Table:**

Bruker APEXII CCD diffractometer	5044 independent reflections
Radiation source: fine-focus sealed tube	4829 reflections with *I* > 2σ(*I*)
graphite	*R*_int_ = 0.016
φ and ω scans	θ_max_ = 27.5°, θ_min_ = 1.6°
Absorption correction: multi-scan (*SADABS*; Sheldrick, 1996)	*h* = −9→9
*T*_min_ = 0.331, *T*_max_ = 0.537	*k* = −16→16
9343 measured reflections	*l* = −17→17

### Refinement

**Table:**

Refinement on *F*^2^	Primary atom site location: structure-invariant direct methods
Least-squares matrix: full	Secondary atom site location: difference Fourier map
*R*[*F*^2^ > 2σ(*F*^2^)] = 0.025	Hydrogen site location: inferred from neighbouring sites
*wR*(*F*^2^) = 0.067	H atoms treated by a mixture of independent and constrained refinement
*S* = 1.04	*w* = 1/[σ^2^(*F*_o_^2^) + (0.0509*P*)^2^ + 0.1124*P*] where *P* = (*F*_o_^2^ + 2*F*_c_^2^)/3
5044 reflections	(Δ/σ)_max_ = 0.001
302 parameters	Δρ_max_ = 3.05 e Å^−^^3^
1 restraint	Δρ_min_ = −1.27 e Å^−^^3^

### Fractional atomic coordinates and isotropic or equivalent isotropic displacement parameters (Å^2^)

**Table:**

	*x*	*y*	*z*	*U*_iso_*/*U*_eq_	
Au1	0.298203 (14)	0.304054 (8)	0.394619 (8)	0.01445 (6)	
Cl1	0.28189 (13)	0.32712 (7)	0.56013 (7)	0.02448 (17)	
Cl2	0.31382 (13)	0.49176 (6)	0.32181 (7)	0.02314 (17)	
Cl3	0.31133 (12)	0.28082 (6)	0.22834 (6)	0.01975 (16)	
Cl4	0.28622 (13)	0.11653 (6)	0.46788 (7)	0.02373 (17)	
O1	0.1850 (4)	0.3950 (2)	0.7782 (2)	0.0258 (5)	
O2	0.3590 (4)	1.22613 (19)	0.9995 (2)	0.0239 (5)	
N1	0.2353 (4)	0.7025 (2)	0.9069 (2)	0.0163 (5)	
H1	0.247 (6)	0.7760 (9)	0.885 (3)	0.029 (11)*	
N2	0.2032 (4)	0.7897 (2)	0.6684 (2)	0.0187 (5)	
N3	0.3136 (4)	0.9265 (2)	0.8548 (2)	0.0156 (5)	
N4	0.1257 (4)	0.8534 (2)	1.0949 (2)	0.0168 (5)	
C1	0.2546 (5)	0.6495 (3)	1.0088 (3)	0.0186 (6)	
H1A	0.2662	0.6912	1.0567	0.022*	
C2	0.2576 (5)	0.5354 (3)	1.0441 (3)	0.0203 (6)	
H2	0.2704	0.4994	1.1159	0.024*	
C3	0.2420 (5)	0.4737 (3)	0.9747 (3)	0.0205 (6)	
H3	0.2447	0.3954	0.9977	0.025*	
C4	0.2222 (4)	0.5296 (3)	0.8712 (3)	0.0164 (6)	
C5	0.2212 (4)	0.6449 (2)	0.8388 (2)	0.0153 (6)	
C6	0.2012 (4)	0.4900 (3)	0.7772 (3)	0.0181 (6)	
C7	0.1980 (5)	0.5922 (3)	0.6874 (3)	0.0182 (6)	
C8	0.1879 (5)	0.6097 (3)	0.5801 (3)	0.0212 (7)	
H8	0.1825	0.5501	0.5504	0.025*	
C9	0.1863 (5)	0.7187 (3)	0.5186 (3)	0.0212 (7)	
H9	0.1809	0.7349	0.4445	0.025*	
C10	0.1924 (5)	0.8047 (3)	0.5642 (3)	0.0223 (7)	
H10	0.1888	0.8781	0.5195	0.027*	
C11	0.2057 (4)	0.6848 (3)	0.7249 (3)	0.0155 (6)	
C12	0.3999 (4)	0.9788 (3)	0.7520 (3)	0.0181 (6)	
H12	0.4178	0.9380	0.7009	0.022*	
C13	0.4636 (4)	1.0882 (3)	0.7171 (3)	0.0180 (6)	
H13	0.5212	1.1206	0.6436	0.022*	
C14	0.4435 (4)	1.1511 (2)	0.7894 (3)	0.0176 (6)	
H14	0.4858	1.2264	0.7675	0.021*	
C15	0.3585 (4)	1.0977 (2)	0.8947 (3)	0.0154 (6)	
C16	0.2951 (4)	0.9874 (2)	0.9225 (2)	0.0139 (6)	
C17	0.3142 (4)	1.1372 (2)	0.9930 (3)	0.0165 (6)	
C18	0.2124 (4)	1.0415 (2)	1.0813 (3)	0.0162 (6)	
C19	0.1345 (4)	1.0290 (3)	1.1887 (3)	0.0181 (6)	
H19	0.1383	1.0876	1.2200	0.022*	
C20	0.0503 (4)	0.9262 (3)	1.2485 (3)	0.0199 (6)	
H20	−0.0062	0.9133	1.3226	0.024*	
C21	0.0490 (4)	0.8422 (3)	1.1996 (3)	0.0195 (6)	
H21	−0.0094	0.7729	1.2426	0.023*	
C22	0.2039 (4)	0.9522 (2)	1.0394 (2)	0.0143 (5)	

### Atomic displacement parameters (Å^2^)

**Table:**

	*U*^11^	*U*^22^	*U*^33^	*U*^12^	*U*^13^	*U*^23^
Au1	0.01648 (8)	0.01391 (8)	0.01188 (8)	0.00013 (5)	−0.00036 (5)	−0.00370 (5)
Cl1	0.0377 (5)	0.0222 (4)	0.0142 (4)	0.0028 (3)	−0.0047 (3)	−0.0066 (3)
Cl2	0.0343 (4)	0.0151 (4)	0.0193 (4)	−0.0012 (3)	−0.0052 (3)	−0.0038 (3)
Cl3	0.0262 (4)	0.0187 (4)	0.0144 (4)	−0.0011 (3)	−0.0020 (3)	−0.0061 (3)
Cl4	0.0333 (4)	0.0151 (4)	0.0194 (4)	0.0019 (3)	−0.0005 (3)	−0.0030 (3)
O1	0.0391 (14)	0.0147 (11)	0.0241 (13)	−0.0037 (10)	−0.0044 (11)	−0.0072 (10)
O2	0.0348 (13)	0.0135 (11)	0.0251 (13)	0.0004 (9)	−0.0077 (11)	−0.0074 (10)
N1	0.0197 (12)	0.0112 (12)	0.0171 (13)	0.0000 (9)	−0.0020 (10)	−0.0038 (10)
N2	0.0205 (13)	0.0146 (12)	0.0195 (14)	−0.0029 (10)	−0.0032 (11)	−0.0030 (10)
N3	0.0171 (12)	0.0149 (12)	0.0136 (12)	−0.0002 (9)	−0.0016 (10)	−0.0033 (10)
N4	0.0180 (12)	0.0155 (12)	0.0162 (13)	−0.0018 (9)	−0.0016 (10)	−0.0048 (10)
C1	0.0229 (15)	0.0171 (14)	0.0161 (15)	−0.0012 (11)	−0.0014 (12)	−0.0068 (12)
C2	0.0255 (16)	0.0161 (15)	0.0177 (16)	0.0011 (12)	−0.0038 (13)	−0.0031 (12)
C3	0.0228 (16)	0.0151 (14)	0.0211 (16)	0.0004 (12)	−0.0026 (13)	−0.0024 (12)
C4	0.0178 (14)	0.0136 (14)	0.0189 (15)	−0.0004 (11)	−0.0024 (12)	−0.0071 (12)
C5	0.0146 (13)	0.0143 (14)	0.0164 (15)	−0.0007 (10)	0.0001 (11)	−0.0054 (12)
C6	0.0189 (14)	0.0171 (15)	0.0191 (15)	0.0002 (11)	−0.0012 (12)	−0.0081 (12)
C7	0.0190 (14)	0.0161 (14)	0.0199 (16)	0.0005 (11)	−0.0021 (12)	−0.0071 (12)
C8	0.0212 (15)	0.0241 (16)	0.0198 (16)	−0.0026 (12)	−0.0037 (13)	−0.0089 (13)
C9	0.0194 (15)	0.0265 (17)	0.0169 (16)	−0.0016 (12)	−0.0025 (12)	−0.0054 (13)
C10	0.0224 (16)	0.0218 (16)	0.0197 (17)	−0.0023 (12)	−0.0048 (13)	−0.0007 (13)
C11	0.0137 (13)	0.0137 (14)	0.0181 (15)	−0.0001 (10)	−0.0023 (11)	−0.0035 (12)
C12	0.0190 (14)	0.0188 (15)	0.0162 (15)	0.0008 (11)	−0.0023 (12)	−0.0056 (12)
C13	0.0184 (14)	0.0176 (14)	0.0141 (14)	−0.0006 (11)	−0.0007 (12)	−0.0002 (12)
C14	0.0169 (14)	0.0130 (13)	0.0214 (16)	−0.0009 (11)	−0.0040 (12)	−0.0024 (12)
C15	0.0156 (13)	0.0128 (13)	0.0177 (15)	0.0032 (10)	−0.0046 (12)	−0.0035 (12)
C16	0.0122 (13)	0.0133 (13)	0.0155 (14)	0.0014 (10)	−0.0027 (11)	−0.0032 (11)
C17	0.0178 (14)	0.0131 (13)	0.0190 (15)	0.0030 (11)	−0.0057 (12)	−0.0040 (12)
C18	0.0149 (13)	0.0157 (14)	0.0183 (15)	0.0011 (11)	−0.0043 (11)	−0.0049 (12)
C19	0.0193 (14)	0.0184 (14)	0.0193 (15)	0.0028 (11)	−0.0060 (12)	−0.0084 (12)
C20	0.0170 (14)	0.0270 (16)	0.0148 (15)	0.0024 (12)	−0.0009 (12)	−0.0064 (13)
C21	0.0181 (14)	0.0172 (14)	0.0206 (16)	−0.0011 (11)	−0.0021 (12)	−0.0025 (12)
C22	0.0120 (12)	0.0149 (13)	0.0165 (14)	0.0020 (10)	−0.0036 (11)	−0.0047 (11)

### Geometric parameters (Å, °)

**Table:**

Au1—Cl1	2.2720 (8)	C7—C8	1.389 (5)
Au1—Cl2	2.2882 (8)	C7—C11	1.405 (4)
Au1—Cl3	2.2864 (8)	C8—C9	1.386 (5)
Au1—Cl4	2.2872 (8)	C8—H8	0.9500
O1—C6	1.208 (4)	C9—C10	1.393 (5)
O2—C17	1.209 (4)	C9—H9	0.9500
N1—C5	1.331 (4)	C10—H10	0.9500
N1—C1	1.355 (4)	C12—C13	1.385 (4)
N1—H1	0.89 (1)	C12—H12	0.9500
N2—C11	1.325 (4)	C13—C14	1.396 (4)
N2—C10	1.355 (4)	C13—H13	0.9500
N3—C16	1.326 (4)	C14—C15	1.381 (4)
N3—C12	1.354 (4)	C14—H14	0.9500
N4—C22	1.326 (4)	C15—C16	1.400 (4)
N4—C21	1.352 (4)	C15—C17	1.498 (4)
C1—C2	1.384 (4)	C16—C22	1.493 (4)
C1—H1A	0.9500	C17—C18	1.500 (4)
C2—C3	1.387 (5)	C18—C19	1.382 (4)
C2—H2	0.9500	C18—C22	1.402 (4)
C3—C4	1.383 (5)	C19—C20	1.392 (4)
C3—H3	0.9500	C19—H19	0.9500
C4—C5	1.397 (4)	C20—C21	1.396 (4)
C4—C6	1.503 (4)	C20—H20	0.9500
C5—C11	1.471 (4)	C21—H21	0.9500
C6—C7	1.486 (5)		
			
Cl1—Au1—Cl3	179.43 (3)	N2—C10—C9	123.9 (3)
Cl1—Au1—Cl4	90.27 (3)	N2—C10—H10	118.1
Cl3—Au1—Cl4	89.73 (3)	C9—C10—H10	118.1
Cl1—Au1—Cl2	89.47 (3)	N2—C11—C7	126.7 (3)
Cl3—Au1—Cl2	90.55 (3)	N2—C11—C5	125.6 (3)
Cl4—Au1—Cl2	179.30 (3)	C7—C11—C5	107.7 (3)
C5—N1—C1	120.1 (3)	N3—C12—C13	123.8 (3)
C5—N1—H1	122 (3)	N3—C12—H12	118.1
C1—N1—H1	118 (3)	C13—C12—H12	118.1
C11—N2—C10	114.0 (3)	C12—C13—C14	120.3 (3)
C16—N3—C12	115.2 (3)	C12—C13—H13	119.8
C22—N4—C21	115.0 (3)	C14—C13—H13	119.8
N1—C1—C2	120.9 (3)	C15—C14—C13	116.1 (3)
N1—C1—H1A	119.6	C15—C14—H14	121.9
C2—C1—H1A	119.6	C13—C14—H14	121.9
C1—C2—C3	120.1 (3)	C14—C15—C16	119.8 (3)
C1—C2—H2	120.0	C14—C15—C17	131.3 (3)
C3—C2—H2	120.0	C16—C15—C17	108.9 (3)
C4—C3—C2	118.0 (3)	N3—C16—C15	124.8 (3)
C4—C3—H3	121.0	N3—C16—C22	126.9 (3)
C2—C3—H3	121.0	C15—C16—C22	108.3 (3)
C3—C4—C5	120.0 (3)	O2—C17—C15	126.5 (3)
C3—C4—C6	132.0 (3)	O2—C17—C18	128.0 (3)
C5—C4—C6	107.9 (3)	C15—C17—C18	105.4 (2)
N1—C5—C4	121.0 (3)	C19—C18—C22	119.4 (3)
N1—C5—C11	129.2 (3)	C19—C18—C17	132.1 (3)
C4—C5—C11	109.8 (3)	C22—C18—C17	108.5 (3)
O1—C6—C7	128.9 (3)	C18—C19—C20	116.5 (3)
O1—C6—C4	126.0 (3)	C18—C19—H19	121.8
C7—C6—C4	105.1 (3)	C20—C19—H19	121.8
C8—C7—C11	118.2 (3)	C19—C20—C21	120.0 (3)
C8—C7—C6	132.5 (3)	C19—C20—H20	120.0
C11—C7—C6	109.4 (3)	C21—C20—H20	120.0
C9—C8—C7	116.6 (3)	N4—C21—C20	124.0 (3)
C9—C8—H8	121.7	N4—C21—H21	118.0
C7—C8—H8	121.7	C20—C21—H21	118.0
C8—C9—C10	120.7 (3)	N4—C22—C18	125.2 (3)
C8—C9—H9	119.7	N4—C22—C16	126.0 (3)
C10—C9—H9	119.7	C18—C22—C16	108.8 (3)
			
C5—N1—C1—C2	−0.8 (5)	C16—N3—C12—C13	−0.8 (5)
N1—C1—C2—C3	0.4 (5)	N3—C12—C13—C14	1.0 (5)
C1—C2—C3—C4	−0.4 (5)	C12—C13—C14—C15	0.1 (5)
C2—C3—C4—C5	0.9 (5)	C13—C14—C15—C16	−1.3 (4)
C2—C3—C4—C6	−179.7 (3)	C13—C14—C15—C17	179.7 (3)
C1—N1—C5—C4	1.2 (5)	C12—N3—C16—C15	−0.5 (4)
C1—N1—C5—C11	−177.8 (3)	C12—N3—C16—C22	179.1 (3)
C3—C4—C5—N1	−1.3 (5)	C14—C15—C16—N3	1.5 (5)
C6—C4—C5—N1	179.2 (3)	C17—C15—C16—N3	−179.3 (3)
C3—C4—C5—C11	177.9 (3)	C14—C15—C16—C22	−178.0 (3)
C6—C4—C5—C11	−1.6 (3)	C17—C15—C16—C22	1.2 (3)
C3—C4—C6—O1	5.2 (6)	C14—C15—C17—O2	−5.5 (6)
C5—C4—C6—O1	−175.3 (3)	C16—C15—C17—O2	175.4 (3)
C3—C4—C6—C7	−176.8 (3)	C14—C15—C17—C18	177.2 (3)
C5—C4—C6—C7	2.8 (3)	C16—C15—C17—C18	−1.9 (3)
O1—C6—C7—C8	−4.5 (6)	O2—C17—C18—C19	3.9 (6)
C4—C6—C7—C8	177.6 (4)	C15—C17—C18—C19	−178.8 (3)
O1—C6—C7—C11	175.0 (3)	O2—C17—C18—C22	−175.3 (3)
C4—C6—C7—C11	−2.9 (3)	C15—C17—C18—C22	2.0 (3)
C11—C7—C8—C9	0.0 (5)	C22—C18—C19—C20	−0.3 (4)
C6—C7—C8—C9	179.4 (3)	C17—C18—C19—C20	−179.5 (3)
C7—C8—C9—C10	−0.7 (5)	C18—C19—C20—C21	0.4 (5)
C11—N2—C10—C9	−0.6 (5)	C22—N4—C21—C20	−0.4 (5)
C8—C9—C10—N2	1.0 (5)	C19—C20—C21—N4	−0.1 (5)
C10—N2—C11—C7	−0.2 (5)	C21—N4—C22—C18	0.5 (4)
C10—N2—C11—C5	178.5 (3)	C21—N4—C22—C16	−178.9 (3)
C8—C7—C11—N2	0.5 (5)	C19—C18—C22—N4	−0.1 (5)
C6—C7—C11—N2	−179.1 (3)	C17—C18—C22—N4	179.2 (3)
C8—C7—C11—C5	−178.4 (3)	C19—C18—C22—C16	179.3 (3)
C6—C7—C11—C5	2.0 (4)	C17—C18—C22—C16	−1.3 (3)
N1—C5—C11—N2	0.0 (5)	N3—C16—C22—N4	0.0 (5)
C4—C5—C11—N2	−179.1 (3)	C15—C16—C22—N4	179.5 (3)
N1—C5—C11—C7	178.9 (3)	N3—C16—C22—C18	−179.5 (3)
C4—C5—C11—C7	−0.2 (4)	C15—C16—C22—C18	0.1 (3)

### Hydrogen-bond geometry (Å, °)

**Table:**

*D*—H···*A*	*D*—H	H···*A*	*D*···*A*	*D*—H···*A*
N1—H1···N3	0.89 (1)	1.88 (1)	2.762 (4)	168 (4)
